# Prediction of Defect Formation during Resin Impregnation Process through a Multi-Layered Fiber Preform in Resin Transfer Molding by a Proposed Analytical Model

**DOI:** 10.3390/ma11102055

**Published:** 2018-10-22

**Authors:** Dong Gi Seong, Shino Kim, Doojin Lee, Jin Woo Yi, Sang Woo Kim, Seong Yun Kim

**Affiliations:** 1 Department of Polymer Science and Engineering, Pusan National University, Busan 46241, Korea; 2 Aircraft Research and Development Division, Korea Aerospace Industries, Gyeongnam 52529, Korea; shino.kim@koreaaero.com; 3 Ceramic Fiber and Composite Materials Center, Korea Institute of Ceramic Engineering and Technology, Gyeongnam 52851, Korea; doojin@kicet.re.kr; 4 Composites Research Division, Korea Institute of Materials Science, Gyeongnam 51508, Korea; yjw0628@kims.re.kr (J.W.Y.); swkim@kims.re.kr (S.W.K.); 5 Department of Organic Materials and Fiber Engineering, Chonbuk National University, Jeonbuk 54896, Korea

**Keywords:** resin transfer molding, fiber preform, defect

## Abstract

It is very important to predict any defects occurring by undesired fiber deformations to improve production yields of resin transfer molding, which has been widely used for mass production of carbon fiber reinforced composite parts. In this study, a simple and efficient analytic scheme was proposed to predict deformations of a multi-layered fiber preform by comparing the forces applied to the preform in a mold of resin transfer molding. Friction coefficient of dry and wet states, permeability, and compressive behavior of unidirectional (UD) and plain woven (PW) carbon fabrics were measured, which were used to predict deformations of the multi-layered fiber preforms with changing their constitution ratios. The model predicted the occurrence, type, and position of fiber deformation, which agreed with the experimental results of the multi-layered preforms.

## 1. Introduction

High speed liquid molding processes, such as high pressure resin transfer molding (HP-RTM), have been successfully applied to the mass production system of automotive lightweight carbon fiber reinforced composite components [1,2,3]. Automated process equipment has been set up and appropriate materials such as carbon fabrics and fast cure type epoxy resin have been developed for automotive mass production systems. At present, it is very crucial to increase the production yield for increasing the amount and area of applications. In the processes, high pressure for both efficient mixing and fast injection of resin is often used and the critical process control is required for successful production of composite parts without defects. Much effort for flow simulation of the resin transfer molding has made it possible to predict some important results such as mold filling time and formation of unfilled area [4,5,6,7,8,9,10]. However, many portions of defects in size, mechanical properties and appearance of the final product are caused by undesirable deformations of fiber preform during the process [11,12,13], which has not been considered in the general mold filling simulations. Some researchers tried to consider the heterogeneity of fiber preform by stochastic methods [14,15,16], which was one of the efficient approaches to predict the undesired defect formation but did not reflect the relating details of competing forces on fiber deformations. Endruweit et al. tried to measure the flow induced fiber deformation and investigated the corresponding mechanism by considering both friction and compressive forces [17]. They measured the cases of multiple layer-stacked fiber preform from one kind of fabric. However, studies on the deformation of multi-layered fiber preforms with multiple kinds of fabric, which are widely used in most industrial composite fabrication processes, are not available. In our previous study, we investigated the resin flow induced deformation of unidirectional carbon fiber preform in the mold filling process by considering friction, in-mold stiffness and shear force by resin flow [18]. Effects of the volume fraction and orientation angle of unidirectional fiber preform were investigated, which mainly influenced on occurrence and type of the fiber preform deformation.

Specific fiber preforms with various laminate structures are used to fabricate structural composite parts for satisfying the required mechanical performances in industrial applications. In general, multi-layered preforms with different texturing structures such as unidirectional non-crimped fabric and plain or twill woven ones are used in most of the composite parts. It is expected that the laminate structure of multi-layered fiber preform strongly influences the occurrence and type of deformation during the manufacturing process. Each set of experiment is necessary to find the optimum process condition preventing defects by the undesired fiber deformation, which is still a difficult and time-consuming process. In this study, an analytical model for predicting deformation of multi-layered carbon fiber preform by using some experimental results of single-layered one was proposed, and the specific cases with unidirectional and plain woven fabric were investigated by comparing the predicted results with the experimental ones. It is expected that the proposed scheme can be used to predict the deformation behavior of any specific stacking combination of fiber preforms by using the experimental results of constituent single layers, which will be very efficient to design the stacking structure of fiber preform with considering manufacturability, preventing the undesired deformation during the resin transfer molding process.

## 2. Materials and Methods

**Figure 1:**
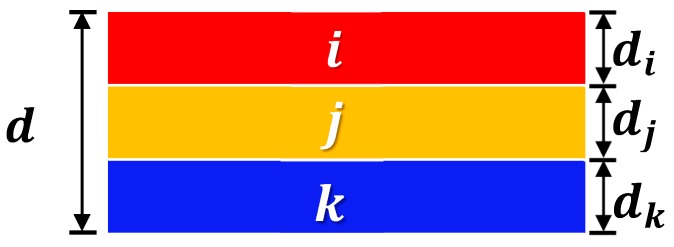
Schematic diagram of the multi-layered fiber preform.

**Figure 2:**
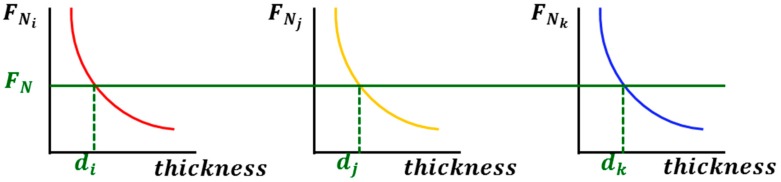
Schematic diagram of compression tests for each fabric layer in the multi-layered fiber preform.

The model evaluation starts from the construction of a general multi-layered fiber preform structure. Schematic diagram of the structure for three different kinds of fiber mats, *i*, *j*, and *k*, is shown in Figure 1. The thickness of the preform (*d*) is obtained by sum of each layer thickness, *d_i_*, *d_j_*, and *d_k_*. The entire preform is compressed by normal force (*F_N_*) which is equally applied to each layer (*F_N__i_*, *F_N__j_*, and *F_N__k_*) because of their serial arrangement along the normal direction to the surface of fabric. Compression behavior of the multi-layered preform can be obtained by measuring the thickness of the individual layer by increasing normal force and combining the resultant graphs, which is described in Figure 2. The compression behaviors of fiber preforms in their compaction [19,20] and resin impregnation processes [21,22] were well described both theoretically and experimentally in previous research. Basic relationships of each layer are expressed in the following Equations (1) and (2).

(1) $$\;F_{N}=F_{N_{i}}=F_{N_{j}}=F_{N_{k}}\;$$

(2) $$\;d=d_{i}+d_{j}+d_{k}\;$$

Average in-plane permeability of the multi-layered preform (*K*) can be predicted by a thickness weighted averaging scheme using the permeability and thickness of each layer, which is written as Equation (3) [23,24].

(3) $$\;K=K_{i}\frac{d_{i}}{d}+K_{j}\frac{d_{j}}{d}+K_{k}\frac{d_{k}}{d}\;$$

where *K_i_*, *K_j_*, and *K_k_* are the in-plane permeabilities of each layer at the specified thickness or volume fraction. Development of injection pressure at a certain flow front can be calculated by Darcy’s law at a constant flow rate condition, from which shear force by resin flow (*F_flow_*) can be calculated by multiplying the injection pressure (*P_in_*) measured during the resin flow with a constant rate and cross-sectional area of fiber preform (*A*) [25,26,27]. We conducted the experiment for flow induced fiber deformation at a constant flow rate condition by using Instron equipped with pneumatic cylinder as described in our previous study [18]. Even though the constant pressure injection is widely used in practical resin transfer molding processes, we chose the injection at a constant flow rate in this experiment because we could efficiently observe the resin flow and the related fiber deformation with increasing injection pressure as flow front advances from one set of experiment. The pressure at the moment when te simple he fiber deformation starts can be regarded as the critical pressure causing the defect in each set of experiments. Thprocedure is shown in Equations (4)–(6).

(4) $$u=-\frac{K}{\eta }\times \frac{0-P_{in}}{x_{f}}$$

(5) $$P_{in}=\frac{\eta x_{f}u}{K}\;$$

(6) $$\;F_{flow}=P_{in}A\;$$

where *u* is the velocity of flow, *x_f_* is the distance between inlet and flow front, and *η* is the viscosity of resin.

**Figure 3:**
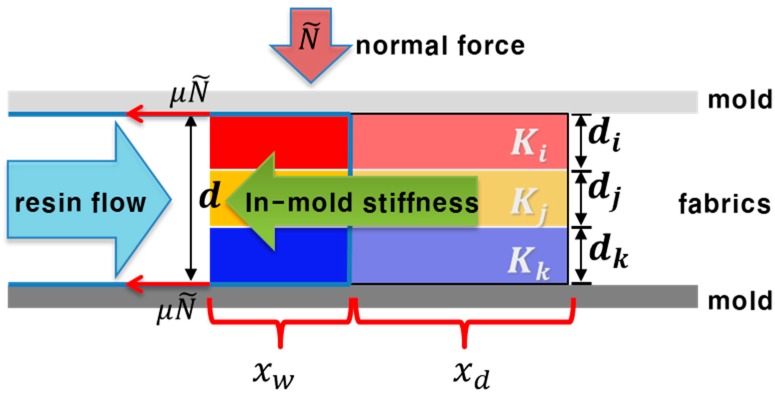
Schematic diagram of the relating forces and parameters during resin impregnation through the multi-layered fiber preform in resin transfer molding process. *x_w_* and *w_d_* are the lengths of wet and dry regions in the fiber preform, respectively.

Friction force (*F_friction_*) between the fiber preform and mold at a certain flow front is calculated by applying a simple rule of mixture on friction coefficient, as shown in Equation (7).

(7) $$\;F_{friction}=2F_{N}\left(\mu_{w}\frac{x_{f}}{x}+\mu_{d}\left(1-\frac{x_{f}}{x}\right)\right)\;$$

where *μ_w_* and *μ_d_* are the friction coefficients at the wet and dry states, and *x* is the total length of fiber preform in the mold. In-mold stiffness of the multi-layered preform at a certain flow front is obtained from the experiment at a constant compression speed or flow rate condition, which was described in our previous research [18]. In this study, the in-mold stiffness of preform was measured in the dry state because we assumed that the initial moment of local deformation occurred in fiber preform was when the fluid reached the fibers in their dry state. The friction force was obtained by considering both dry and wet components of fiber preform because it was assumed that preform slip was occurred from the competition between flow force of resin and the total resultant friction force at an instant. The overall relating forces and parameters during resin impregnation through the multi-layered fiber preform in resin transfer molding are represented schematically in Figure 3.

As a result, it is possible to compare the magnitude of three related forces by friction, in-mold stiffness and resin flow as flow advances through the multi-layered fiber preform, from which we can predict the deformability and the type of deformation of the preform at a specific time.

## 3. Experiments

Multi-layered fiber preforms were prepared from the combination of unidirectional (UD, PANEX 35 50K UD150 from Zoltek, St. Louis, MO, USA). with transverse orientation to flow (90° to the flow direction) and plain woven (PW, TR 30 from Mitsubishi, Tokyo, Japan) carbon fabrics. Basic properties of the fabrics are summarized in Table 1. Silicon oil from Shinetsu (KF-96H-350CS, Tokyo, Japan) was used for test fluid. Three combinations of the multi-layered preform were set up by increasing the ratio of PW to UD fabric from 25% to 75% while maintaining total fiber volume fraction of 50%, which were selected to possibly observe various kinds of deformation such as slip and wrinkle by changing the ratio. The basic assumption in this study is that deformation of the fiber preform is determined by comparing the magnitude of acting forces to the fiber preform. For example, rigid body deformation such as slip of fiber preform along the mold surface can occur if flow force is larger than the total friction force between fiber and mold. Local deformation can occur if flow force is larger than force by in-mold stiffness even though it is smaller than total friction force. Wrinkle is a typical example of the local deformation, at which the front part of fabric is deformed to make a wrinkle even though the rear part is not deformed [18]. It can be assumed that local deformation such as wrinkle is easily occurred in UD fabric with a transverse orientation to the resin flow because of its low in-mold stiffness, and resistance to local deformation is increased by increasing the ratio of PW fabric. The detail composition of multi-layered preform is shown in Table 2. Numbers of each layer are determined from a specified ratio while total thickness of the multi-layered preform is fixed to 6 mm and the resultant fiber volume fraction of the multi-layered preform is approximately 50%.

Three kinds of tests were performed for unit fabrics which constitute the multi-layered preform. Coulomb and hydrodynamic friction coefficients at dry and wet states were measured for transversely oriented UD and PW fabrics by using the equipment described in our previous research [18]. Compression tests for two kinds of fabrics based on ASTM D5729 were performed by Instron 5882 to obtain the change in normal force with respect to thickness of the fabric which is schematically shown in Figure 2 [28]. In-plane permeability of each fabric was also measured at several fiber volume fractions by radial flow experiment [29,30,31]. The measured values are used to predict forces by friction and resin flow of the multi-layered fiber preform with respect to flow front position through the analytical scheme as described in the previous section.

In-mold stiffness of the multi-layered fiber preform was measured for three combinations by using a specific mold in the universal test machine which was described in the previous study [18]. The magnitude of force by in-mold stiffness of the multi-layered preform was compared with the predicted values of force by friction and resin flow, from which we could predict the occurrence and type of deformation in the preform. Finally, flow induced deformation of the multi-layered preform was observed by using the measurement system in the resin transfer molding process. The analytical model was verified by comparing the predicted deformation results with the experimentally measured ones for the multi-layered fiber preform. We comment on repetition of each measurements and the related deviations, but plot the most stable result instead of the average values in Figures 7, 8, 10 and 11 to prevent the readers from misunderstanding the results by the instability and scattering.

## 4. Results

### 4.1. Friction Force

**Figure 4:**
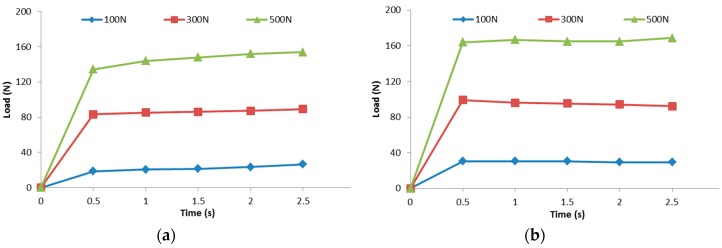
Friction forces between dry fabric and mold at three kinds of applied normal forces (100 N, 300 N, and 500 N): (**a**) transversely oriented UD fabric; and (**b**) PW fabric.

**Figure 5:**
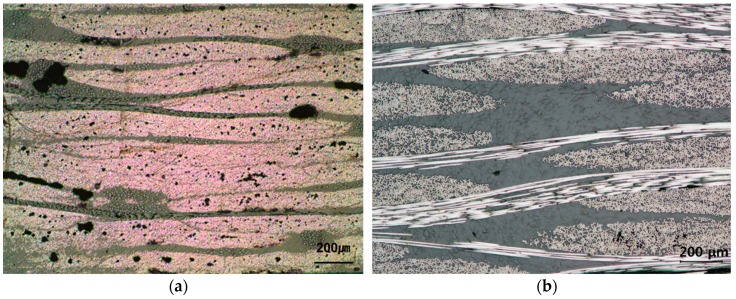
Cross-sectional microscope images of the carbon fiber reinforced composite specimens: (**a**) UD fabric; and (**b**) PW fabric.

Experimental results of the friction forces for transversely oriented dry carbon UD and PW fabrics are plotted in Figure 4. Friction forces at a constant moving speed of 0.01 m/s are plotted with time at normal forces of 100, 200 and 300 N. Coulomb friction coefficient was determined at the inflection point of plot through dividing the measured friction force by the applied normal force. Coulomb friction coefficients of the dry fabrics are summarized in Table 3. The resultant friction coefficient averaged from three repeated measurements of transversely oriented UD fabric was 0.130 (±0.012) and that of plain woven fabric was 0.160 (±0.008). It is thought that the plain woven fabric has the higher friction coefficient than the transversely oriented UD fabric because of its more tortuous and complex geometry of the carbon fiber, as shown in the cross-sectional microscope images of PW and UD carbon fiber reinforced composite specimens in Figure 5. It is shown that PW fabrics have wavier surfaces with the higher roughness because they are woven at a ratio of 1:1 along weft and warp directions, while UD fabric have nearly flat surfaces because they have only a small amount of polyester stitch yarn across the unidirectional carbon fibers.

**Figure 6:**
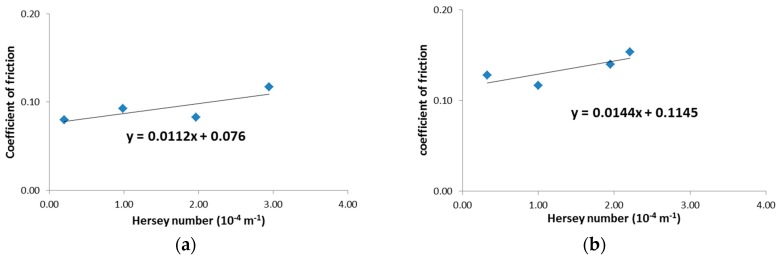
Hydrodynamic friction coefficient as a function of Hersey number: (**a**) transversely oriented UD fabric; and (**b**) PW fabric.

Hydrodynamic friction coefficients of the same fabrics in their wet state were also measured with respect to the Hersey number, as plotted in Figure 6, and the regression values from measurements at four Hersey numbers are summarized in Table 3. Typical hydrodynamic lubrication behavior between the friction coefficient and the Hersey number was observed at both fabric structures, in which the hydrodynamic friction coefficient increases with increasing the Hersey number in the hydrodynamic lubrication region after it decreases in the elasto-hydrodynamic lubrication region with the lower Hersey number [32]. Friction coefficients of wet fabrics were much smaller than those of dry fabrics for both structures, which might be caused by the lubrication effect of liquid resin.

**Figure 7:**
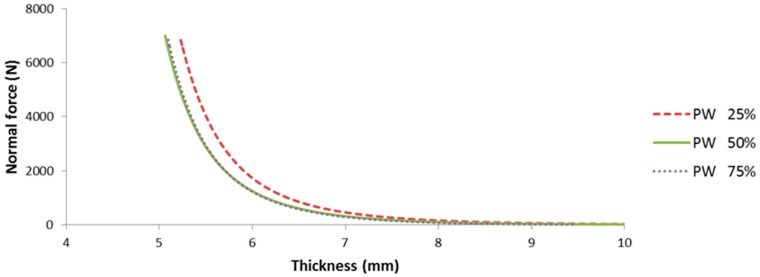
Change in normal forces of the multi-layered fiber preform with respect to the thickness by compressive force with varying ratio of PW to UD from 0% to 100%.

**Figure 8:**
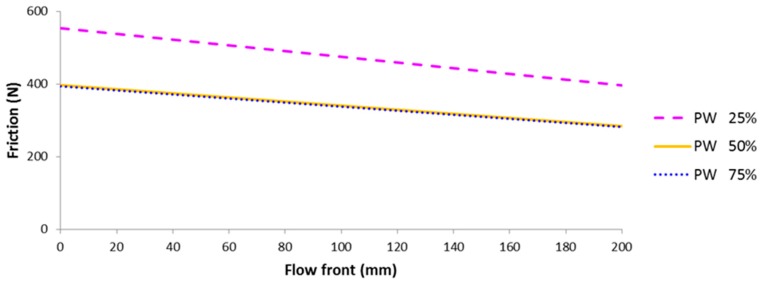
Predicted friction force with respect to flow front position in the multi-layered preform with various ratios of PW to UD carbon fabric.

Friction force of a multi-layered fiber preform can be calculated at any time with an instantaneous flow front position and a specified normal force. At first, compression tests were performed for each layer constituting the multi-layered preform, from which normal force can be determined at the specified thickness of 6 mm, as shown in Figure 7. The resultant friction coefficient at a specific flow front could be calculated from the simple rule of mixture shown in Equation (7) using the dry and hydrodynamic friction coefficients and the corresponding normal force determined by the result of Figure 7, as plotted in Figure 8. The total friction force is decreased as the flow front advances because the contribution of hydrodynamic friction coefficient becomes larger. One of interesting things is that the friction force becomes larger as the ratio of UD fabric is increased (or PW fabric is decreased) despite the smaller value of friction coefficient in the UD fabric. This is because the UD fabric has a larger normal force than PW at the same thickness, as shown in Figure 7. It might be originated from the tighter structure of UD fabric with the lower number of yarn per bundle (K), FAW and thickness, as shown in Table 1. The slope is slightly steeper as ratio of UD fabric is increased, which is caused by the larger difference between the friction coefficients of dry and wet states in the UD fabric.

### 4.2. In-Mold Stiffness

**Figure 9:**
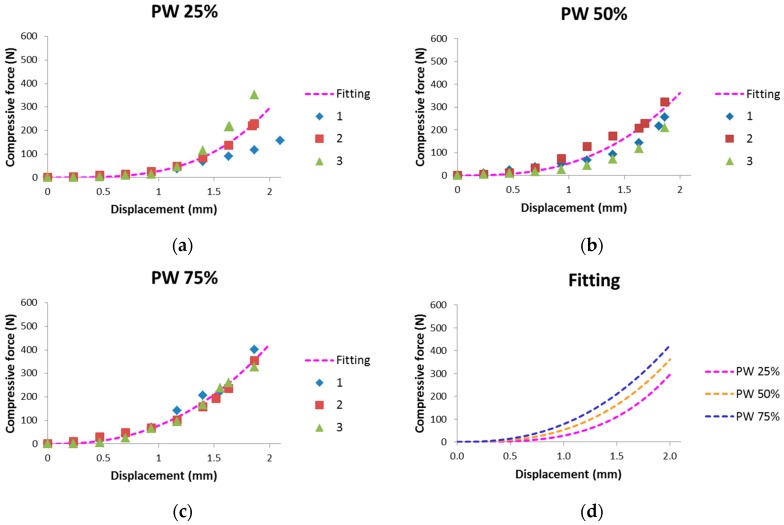
Compressive force with respect to displacement in the in-mold stiffness measurement of the multi-layered fiber preforms with various ratios of PW to UD carbon fabric: (**a**) PW 25%; (**b**) PW 50%; and (**c**) PW 75%; and (**d**) force by in-mold stiffness of the multi-layered preforms with respect to displacement.

Results of in-mold stiffness measurement for the multi-layered fiber preforms with three different ratios of PW to UD carbon fabrics are plotted in Figure 9. Each measurement was repeated three times and the corresponding results are plotted as 1, 2, and 3 in Figure 9a–c. Multi-layered preform with the higher portion of PW has the larger compressive force because compressive force of the UD fabric is smaller than that of PW. It may be caused by weak resistive force of UD fabric due to the transverse orientation to compressive displacement, in which only a small amount of stitching fiber plays a role of resisting the compression.

### 4.3. Flow Induced Deformation of the Multi-Layered Fiber Preform

**Figure 10:**
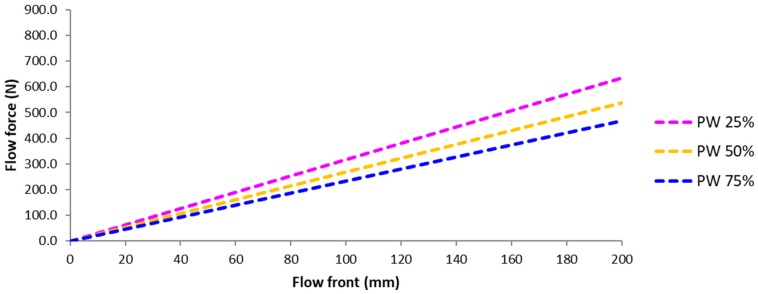
Predicted flow force with respect to flow front position in the multi-layered preform with various ratios of PW to UD carbon fabric.

Main driving force of fiber deformation in the resin impregnation process of liquid composite molding is the pressure (or force) by resin flow. Force by resin flow could be predicted by Darcy’s law using the averaged permeability at a constant flow rate condition, as shown in Equations (4)–(6). The predicted flow force with respect to flow front position in the multi-layered preform with three different ratios of PW to UD carbon fabrics is plotted in Figure 10. Flow force is decreased as the portion of PW fabric is increased because PW fabric has a higher permeability than UD. The permeabilities measured by an unsaturated radial flow method were $1.65\left(\pm 0.09\right)\times 10^{-10}\;m^{2}$ for PW fabric and $4.99\left(\pm 0.29\right)\times 10^{-11}{\;m}^{2}$ for the transverse direction of UD, which were averaged from five measurement values.

**Figure 11:**
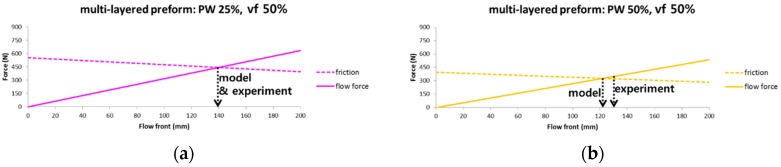

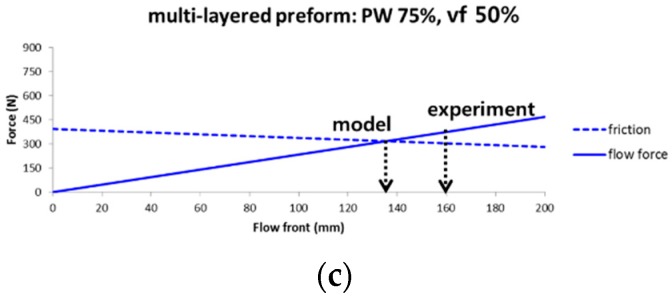
Change of friction and flow force with respect to flow front advancement. Arrows represent slip occurring points predicted by model and measured by experiment. (**a**) PW 25%; (**b**) PW 50%; (**c**) PW 75%.

It is possible to predict the occurrence, type and position of fiber deformation during the RTM process by comparing each force during the flow front advancementin Figure 8, Figure 9 and Figure 10. The calculated flow and friction forces are plotted with respect to flow front position for three kinds of multi-layer composition in Figure 11, and the corresponding experimental results are shown in Table 4. Two colors are used to identify whether the specific fiber deformation occurs in Table 4. A blue colored box indicates no occurrence of the deformation in the condition while a red colored box indicates occurrence of the deformation. A friction force is larger than flow force at the early stage of flow front; the former is decreased, while the latter is increased as flow front advances. The point that flow force starts to be larger than friction, in other words, the intersection of two plots, is the starting position of fiber deformation. The predicted and measured points for fiber deformation in three kinds of multi-layered preforms are identified by arrows, respectively, in Figure 11a–c, which shows that the predicted values by the analytic model agree relatively well with the experimental ones. In-mold stiffness force is not shown in the graphs because it is larger than the other kinds of forces at most positions. It is expected that local deformation, such as wrinkle, occurs when flow force is larger than friction force but less than in-mold stiffness force. Table 4 shows that there are no local deformations in the experiments because in-mold stiffness force is much larger than friction and flow forces.

## 5. Discussion

An analytical model was proposed to predict the occurrence, type and position of fiber deformation during the mold filling stage of liquid composite molding process using a multi-layered fiber preform. Two kinds of processing parameters, friction coefficients in dry and wet states and permeability of every constituent fiber mats in the multi-layer preform, were measured to be used for the model prediction. Thickness variations by applying the compressive forces onto the fiber mat were also measured to calculate the thickness portion of each fiber mat of the multi-layered preform laminated inside the closed mold. The occurrence and position of slip could be predicted by comparing the resultant friction and flow forces at a specific flow front, which was calculated by velocity equation from Darcy’s law. The occurrence and position of local deformation such as wrinkle could also be predicted by comparing the force by in-mold stiffness of fiber preform with the friction and flow forces.

In summary, the analytical scheme was proposed to predict detailed information of realistic fiber deformation, not by doing real liquid molding process but by measuring only some process parameters of fiber mats constituting the used multi-layered preform. It is expected to be used to predict important defects by the deformations of multi-layered fiber preforms in popular high speed liquid composite molding processes such as high pressure resin transfer molding and wet compression molding, which are crucial technologies for mass production of automotive composite parts.

## Figures and Tables

**Table 1:** Basic properties of the used carbon fabrics.

Structure	Supplier	Model	Yarn (K)	FAW* (g/m^2^)	Thickness (mm)
UD	Zoltek	UD150	50	182	0.21
PW	Mitsubishi	TR30M	3	200	0.23

**Table 2:** Detail composition of the multi-layered preforms with varying ratio of PW fabric. *(FAW) Fiber Areal Weight.

Ratio of PW (%)		25 (27.6) *	50	75 (71.4) *
Number of layer	UD	21	14	8
	PW	8	14	20

* Value in bracket is an estimated one using given number of each layer.

**Table 3:** Friction coefficients of unidirectional and plain woven carbon fabrics in their dry and wet states.

Fabric		UD	PW
Friction coefficient	Dry fabric (with standard deviation)	0.130 (±0.012)	0.160 (±0.008)
	Wet fabric (regression value)	0.076	0.115

**Table 4:** Summary of fiber deformation and the magnitude of the related forces.

Type of Deformation (Occurrence of Deformation, O/X)	PW 25%				PW 50%				PW 75%			
	Friction Force	Flow Force (Model)	Flow Force (Exp.)	In-Mold Stiffness Force	Friction Force	Flow Force (Model)	Flow Force (Exp.)	In-mold Stiffness Force	Friction Force	Flow Force (Model)	Flow Force (Exp.)	In-Mold Stiffness Force
-	(N)	(N)	(N)	(N)	(N)	(N)	(N)	(N)	(N)	(N)	(N)	(N)
Local (X)	553	0.880	-	33.3	396	0.520	-	52.8	393	0.369	-	77.9
Rigid body (O)	443	444	495	>1000	323	349	346	>1000	304	375	300	>1000
